# Supplementary material for: Understanding Braess’ Paradox in power grids
Source: Nat Commun. 2022 Sep 14;13:5396. doi: 10.1038/s41467-022-32917-6 (PMC9474455; doi:10.1038/s41467-022-32917-6)
Supplement: Supplementary file 1 — Supplementary Information [file 41467_2022_32917_MOESM1_ESM.pdf]

Supplementary Information accompanying the manuscript Understanding Braess' Paradox in power grids

Benjamin Schäfer°,[1,2,3,4,*] Thiemo Pesch°,[5] Debsankha Manik°,[4,6] Julian Gollenstede,[7] Guosong Lin,[7] Hans-Peter Beck,[7] Dirk Witthaut°,[8,9] and Marc Timme°[4,6,10,†]

[1]*Institute for Automation and Applied Informatics, Karlsruhe Institute of Technology, 76344 Eggenstein-Leopoldshafen, Germany*
[2]*Faculty of Science and Technology, Norwegian University of Life Sciences, 1432 Ås, Norway*
[3]*School of Mathematical Sciences, Queen Mary University of London, United Kingdom*
[4]*Chair for Network Dynamics, Center for Advancing Electronics Dresden (cfaed) and Institute for Theoretical Physics, Technical University of Dresden, 01062 Dresden, Germany*
[5]*Forschungszentrum Jülich, Institute for Energy and Climate Research - Energy Systems Engineering (IEK-10), 52428 Jülich, Germany*
[6]*Network Dynamics, Max Planck Institute for Dynamics and Self-Organization (MPIDS), 37077 Göttingen, Germany*
[7]*Clausthal University of Technology Institute of Electric Power Technology (IEE) Clausthal-Zellerfeld, Germany*
[8]*Forschungszentrum Jülich, Institute for Energy and Climate Research - Systems Analysis and Technology Evaluation (IEK-STE), 52428 Jülich, Germany*
[9]*Institute for Theoretical Physics, University of Cologne, 50937 Köln, Germany*
[10]*Lakeside Labs, Lakeside B04b, 9020 Klagenfurt, Austria*

Within this Supplementary Information, we provide additional evidence and material supporting the narrative and conclusions of the main text. In particular, we provide further details on the laboratory setup, show how increasing the number of lines also results in Braess' paradox, provide additional details on the predictor for Braessian links, its algorithmic implementation and its evaluation statistics.

* email: benjamin.schaefer@kit.edu
† email: marc.timme@tu-dresden.de

Supplementary Note 1

Technical details of the laboratory

The experiments were carried out in the distribution grid laboratory of the "Energieforschungszentrum Niedersachsen (EFZN)" in Goslar, Germany. The technical setup is shown in Supplementary Fig. 1: The four nodes, i.e. two generators and two motors are arranged in a cycle graph. All lines are monitored in terms of the voltage and current over time and resistance R and reactance X can be tuned by using switches, see Supplementary Fig. 2. The overall resistance and reactance per line is slightly different for each of the three phases a, b, c, see Supplementary Table I for all possible values. Most experiments used the minimal resistance of $R \approx 0.18\Omega$ and the maximum inductance $X \approx 0.25\Omega$. This lead to minimal reactive and maximal active power transmission.

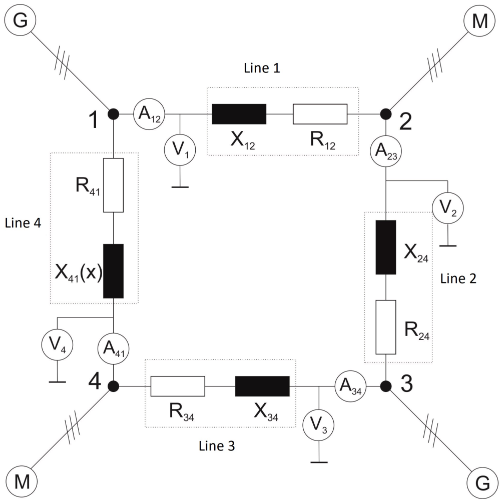

Supplementary Figure 1. Technical scheme of the experimental topology. G: Generator, M: Motor. Both resistance R and reactance X are tuneable on each line. We monitor the voltage (V) at each node and current (A) on each line.

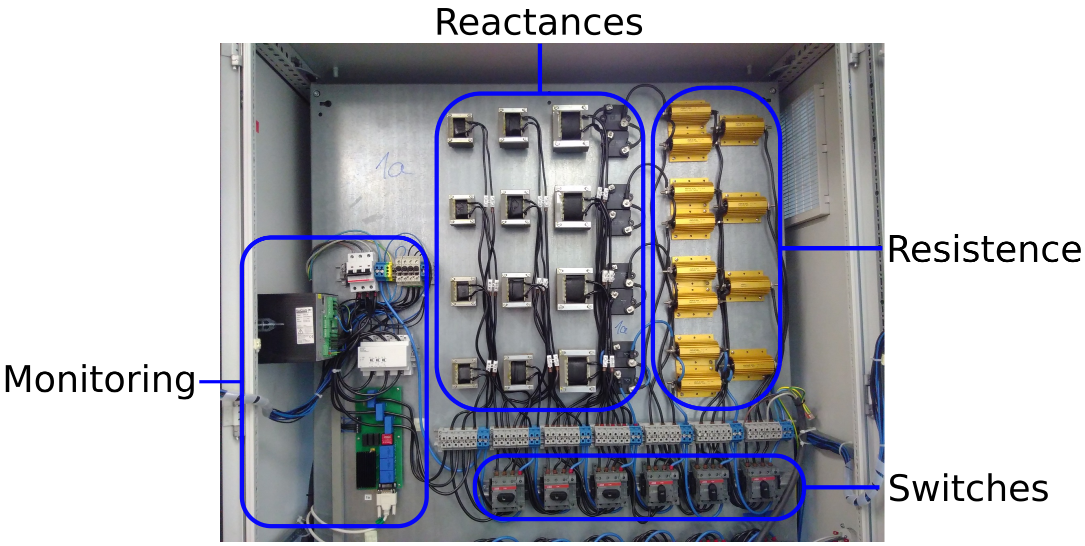

Supplementary Figure 2. Picture of the line elements used for the experiments: Resistance R and reactance X are tuneable on each line by changing the state of the switches. Changes of the line properties are possible while the grid is operating. See Supplementary Table I for possible line parameters.

Of the four nodes of the experiment, two were synchronous machines, which were excited by asynchronous motors (Supplementary Fig. 3). One node was a virtual synchronous machine [1] (Supplementary Fig. 4) and one was a

Supplementary Table I. Line parameters (resistance R and reactance X) for all three phases, with phases labeled a, b, c. R_0 and X_0 give the base line resistance and reactance, respectively, while X_1, X_2, X_3 are the reactances that can be added individually via the switches. $X_{\max}$ then gives the maximum reactances achievable with the setup. Experiments were initiated with all phases and lines as symmetric as possible with their respective R_0 and $X_{\max}$. Next, all possible configurations of the switches were realized to slowly reduce the reactance on one line, while keeping all phases as symmetric as possible.

Line and phase	R_0 in Ω	X_0 in Ω	X_1 in Ω	X_2 in Ω	X_3 in Ω	$X_{\max}$ in Ω
1a	0.177	0.0089	0.0312	0.0597	0.1587	0.2531
1b	0.183	0.0106	0.0303	0.0604	0.1516	0.2454
1c	0.176	0.0090	0.0323	0.0593	0.1485	0.2446
2a	0.180	0.0084	0.0317	0.0603	0.1592	0.2537
2b	0.174	0.0086	0.0324	0.0625	0.1536	0.2475
2c	0.176	0.0084	0.0329	0.0599	0.1491	0.2453
3a	0.176	0.0091	0.0310	0.0596	0.1585	0.2530
3b	0.175	0.0092	0.0317	0.0618	0.1530	0.2468
3c	0.178	0.0093	0.0320	0.0590	0.1483	0.2444
4a	0.175	0.0087	0.0314	0.0599	0.1588	0.2533
4b	0.171	0.0087	0.0322	0.0623	0.1534	0.2473
4c	0.176	0.0088	0.0325	0.0595	0.1488	0.2449

network simulator, the ACS, technically acting similar to a virtual syncrhonous machine (Supplementary Fig. 5).

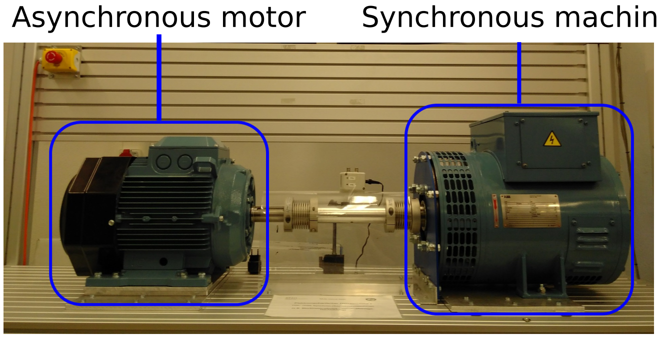

Supplementary Figure 3. The synchronous machine generates power due its physical coupling to an asynchronous/induction motor. The motor is power by the local distribution power grid, while the synchronous machine acts as one node within the laboratory grid.

Uncertainty estimation

Neither the laboratory experiments nor the derived theoretical results are free of uncertainties. Indeed several effects contribute to these uncertainties: While the voltage signal recorded in the laboratory follows a clear sine wave, the current measurement is much noisier, leading to natural uncertainties in the precise current amplitude and phase, thereby adding uncertainty to the transmitted power at each line. An example current trajectory with a sine function fitted to it is displayed in Supplementary Fig. 7.

Furthermore, inspecting the recorded values of the resistance and specifically the inductance in Supplementary Table I on the three different phases for each line, we notice already relative uncertainties of order $\frac{\Delta X}{X} \sim 0.10...0.20$. Since we use simplified one phase calculations, averaging over the different line parameters and thereby currents on each line also increases the uncertainties.

Overall, based on careful comparison of several experimental realizations, we estimate the measured current uncertainty to be $\Delta I_{measured} \sim 0.15\ A$, while the calculated currents are estimated to have an uncertainty of $\Delta I_{calculated}/I \sim 0.05$, mainly due to asymmetric line properties of the phases.

In our calculations we assumed that the power P and the reactive power Q at each node stays constant throughout the experiment. This is likely not 100% the case, leading to additional small deviations. Finally, we note that the

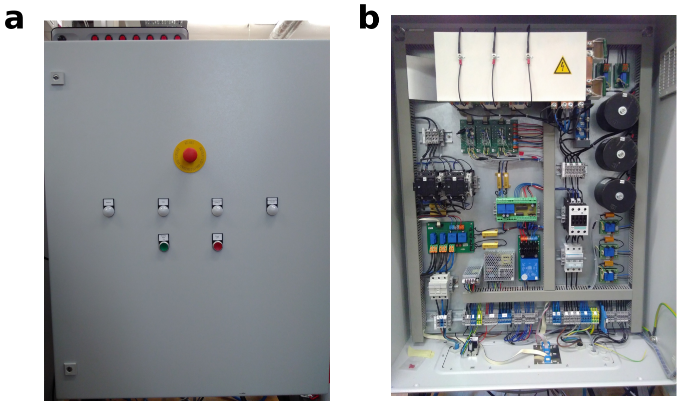

Supplementary Figure 4. A virtual synchronous machine (VISMA) is based on inverter, simulating the effective dynamics of physical synchronous machines [1]. a: The VISMA closed, as used during the experiment with basic manual controls available. b: The VISMA open, allowing a view on the inverters, capacitances and coils. The computer controlling the parameters and VISMA dynamics during the experiment is not shown.

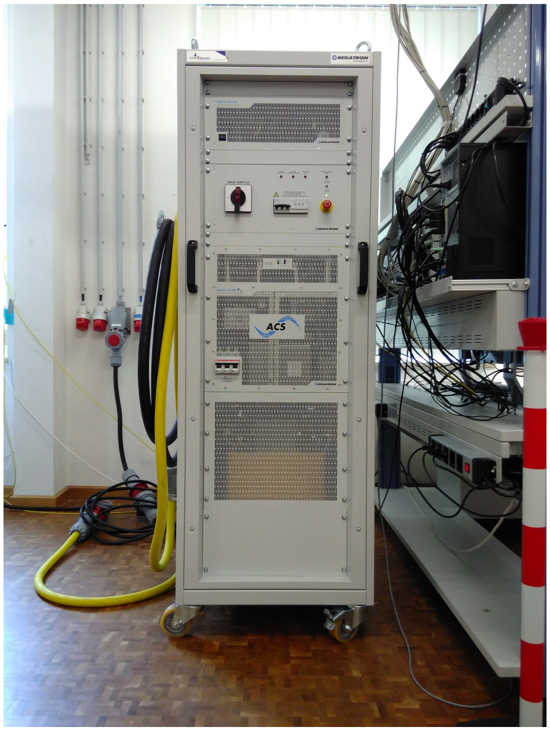

Supplementary Figure 5. The net simulator ACS provides a constant 50Hz frequency to the test grid and was mainly used to stabilize the test grid when changing the line parameters. Its static properties are comparable to other inverter-based technologies, such as the VISMA.

additional inductances introduced in the system likely also increased the resistance of the line. Therefore, reducing the reactance also reduces the resistance and explains why the measured currents in Fig. 2 of the main text exceed the computed ones (which assumed a constant resistance for simplicity).

Concluding, while the uncertainties in the experimental setup are not negligible, we can quantify most effects and all observed trends and predictions are within uncertainty limits.

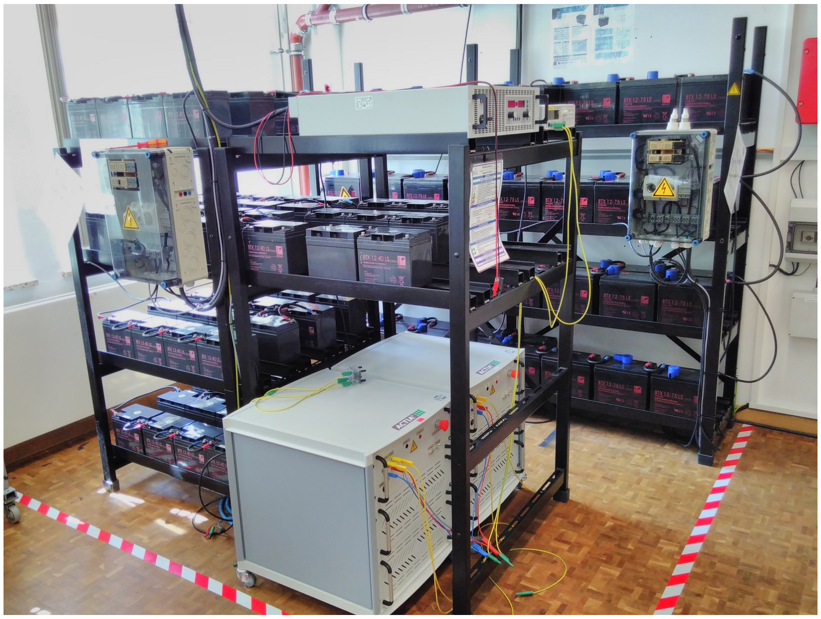

Supplementary Figure 6. The battery stack powers the inverter of the virtual synchronous machine (VISMA).

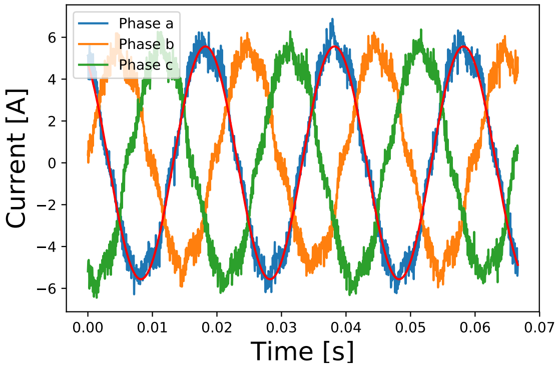

Supplementary Figure 7. Trajectories of the line currents I are noisy and phases are not perfectly symmetric. We display the currents on the three phases of line 1 I_{1a}, I_{1b}, I_{1c}. For phase a, we also display the best fitting sine curve as a red curve. We notice both noisy behavior of the current with respect to the fit and asymmetries between the phases. For example, the green curve of phase c reaches lower values than the orange curve of phase b does.

Supplementary Note 2

Increasing the number of lines and inverting the roles of machines and generators

In the main text, we discussed Braess' paradox in the distribution power grid when upgrading an individual line. Instead of only changing the reactance of one line, we might as well deploy an additional parallel line. We determine the changes in the current when copying line 4, while setting the parameters of all lines (including the copies of line 4) to maximal reactance X and minimal resistance R. Again, the current on line 2 increases, as the number of lines at line 4 increases, see Supplementary Fig. 8. Due to physical restrictions in the laboratory, three parallel lines were only simulated but for one and two parallel lines the agreement between theory and computation is convincing. We also test how robust or results are by running the synchronous machines once as generators (left) and once as motors (right).

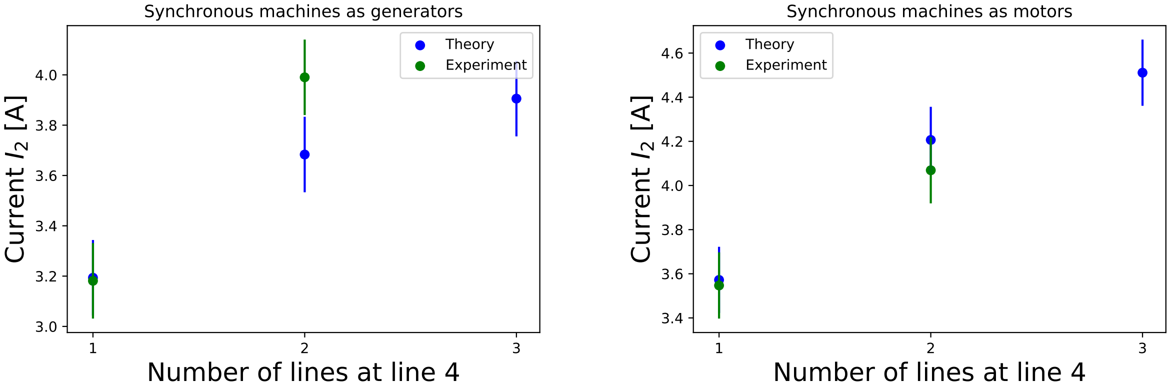

Supplementary Figure 8. Braess' paradox occurs in AC grids when adding parallel lines. We display the current amplitude on line 2 of the network as a function of the how many (parallel) lines are used on line 4. Green dots indicate measurements and blue dots give the theoretical predictions. Left: Synchronous machines run as generators. Right: Synchronous machines run as motors. Increasing the number of lines at line 4 increases and potentially overloads line 2, as seen by the increasing current. Error bars give one standard deviation around the mean value, based on measurement and estimation uncertainties.

Complementing the figures from the main text, we display the current on all lines in Supplementary Fig. 9. As in Supplementary Fig. 8, we operate the synchronous machines once as generators (left) and once as motors (right). In all cases, we observe the expected behavior: The upgraded line 4 but also the unchanged line 2 show an increasing current with reduced reactance, i.e. the highest load increases.

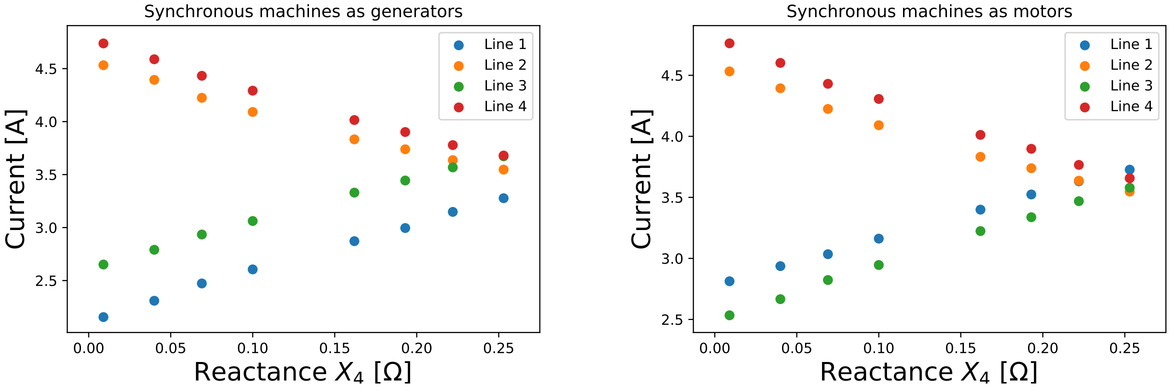

Supplementary Figure 9. Braess' paradox occurs also when reversing the role of generators and consumers in the network. a: The synchronous machines are runs as generators with the VISMAs acting as consumers (motors). b: All lines have comparable current for the maximum reactance $X_4 \approx 0.25\Omega$. Decreasing the reactance X, i.e., increasing the susceptance B increases the currnet on lines 2 and 4, while it decreases the current on lines 1 and 3.

Supplementary Note 3

Rerouting pathways as predictor for Braess' Paradox

In this section we explain in detail the Braess' paradox predictor we demonstrated in Figure 3 of the main text. We explored this topic in greater detail in [2] and [3, Ch. 6].

Definition of Braess' Paradox

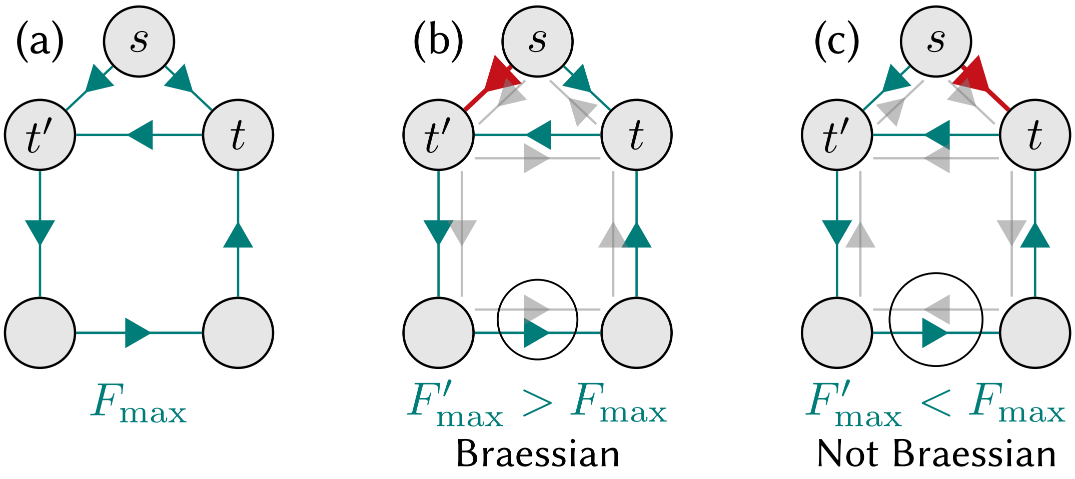

Supplementary Figure 10. Definition of Braessian edges. Green arrows denote direction of steady flows, grey arrows denote direction of flow changes when one edge is enhanced. (a) An example 5 node DC power flow network. Maximum flow is across the bottom edge. (b) Top left edge (red) enhanced. Maximum flow increases (gray arrow and green arrow in same direction). Top left edge is Braessian. (c) Top right edge (red) enhanced. Maximum flow decreases. Top right edge is not Braessian.

We say an edge in a flow network is Braessian if infinitesimally enhancing it (i.e. increasing its capacity) leads to an *increase* in the maximum flow. We illustrate this in Supplementary Fig. 10, where we have an example 5-node DC flow network. Its steady flows are directed as per the green arrows. The maximum flow bearing edge (maxflow edge) is at the bottom. On infinitesimally enhancing the top left edge, the (infinitesimal) flow changes that are caused are directed as per the grey arrows. At the bottom edge (maxflow edge), the flow change (grey arrow) is *in the same direction* as the original flow (green arrow). This means the top left edge is Braessian. The opposite happens in case of the top right edge, hence it is not Braessian.

Therefore, predicting which edges are Braessian is equivalent to answering the following question: On enhancing which edges, will the change in the maximum flow be in the same direction as the the original maximum flow?

Dipole flow equivalence

Notably, the flow changes on infinitesimally enhancing an edge in a DC power flow network are exactly equal to the flows in the same network, if all the power in/outputs are removed and only a single dipole current source of a suitable strength were placed across the edge being enhanced. We illustrate this in Supplementary Fig. 11. For details, we refer the reader to [2, 3].

Detecting BE via dipole sources

We now come back to our quest of predicting which edges are Braessian, i.e. which edges, when enhanced, will cause the flow change at the maxflow edge to be in the same direction as the original flow. The recipe, as per Supplementary Fig. 11, should be to place a dipole across each edge and see if the resulting dipole flow across the maxflow edge aligns

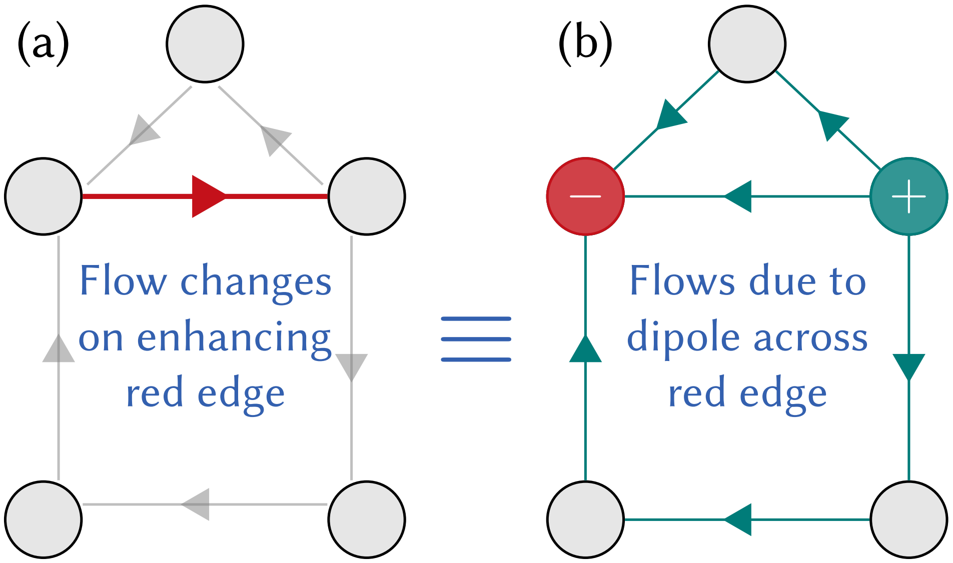

Supplementary Figure 11. (a) Flow changes in a DC power flow network due to infinitesimally enhancing the red edge, are exactly equal to (b) the power flows in the identical network, but all nodal power inputs/outputs set to zero, except a a single dipolar input across the red edge. Note: the equality does *not* hold exactly at one edge: The edge being enhanced.

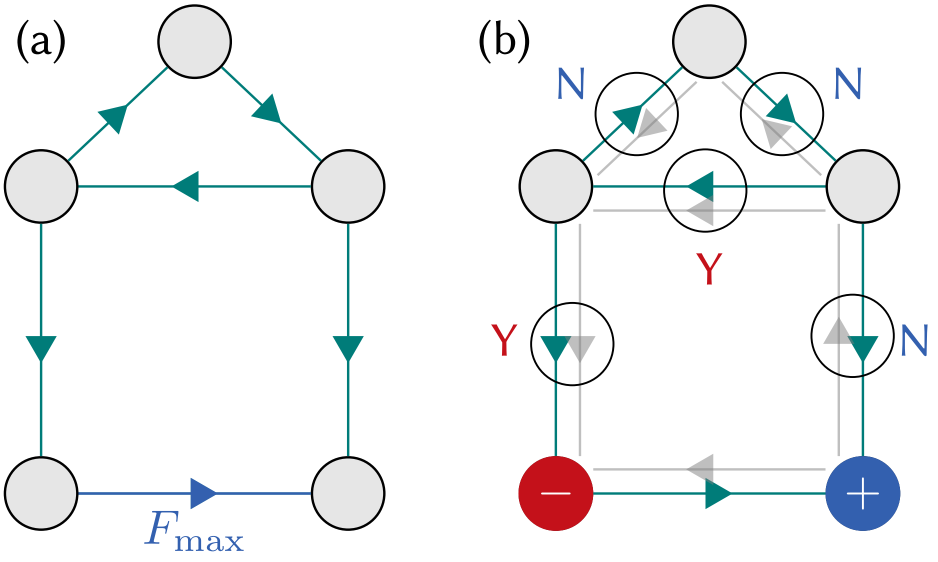

Supplementary Figure 12. Detect all Braessian edges in a network using the dipole equivalence. (a) DC power flows (green arrows) in a network. Maximum flow across the bottom edge. (b) We take a network with identical topology but no nodal power inputs. Place a dipole across the bottom edge and look at the flows (gray arrows). Those edges are Braessian, across which the gray and green arrows are in the same direction.

with the original flow there. Still, we can do even better: We do not need to place a dipole at each edge, we can do it just with one dipole. This is because of a symmetry in the flows due to a dipole source in a network, namely the flow across edge e due to a dipole placed across edge f is identical to the flow across edge f due to a dipole across edge e. This is the discrete analog of the fact that the field due to an electrostatic dipole is symmetric with respect to swapping the location of the dipole and the point at which the field is measured. Again, we refer to [2, 3] for details.

This leads to a short algorithm for determining which edges are Braessian in a network, as we demonstrate in Supplementary Fig. 12. We place a dipole across the maximum flow, in the *opposite direction* to it. The edges at which the resulting dipole flows are in the same direction as the original flows are Braessian. The other flows are not. We formalize this in the following algorithm:

Algorithm 1: Identify Braessian edges

Data: $G(V, E)$, a graph with edge set E and vertex set V.
$\vec{I}$, the power inputs at each node $v \in V$.
$\vec{F}$, the power flows at each edge $e \in E$.
The maximum flow $F_{\max}$ is across edge (s, t), from s to t.
Result: Set of Braessian edges E_{be}, set of non-Braessian edges E_{nbe}.

```
/* start of the algorithm                                                */
```

$E_{\mathsf{be}} \leftarrow \{\}$;
$E_{\mathsf{nbe}} \leftarrow \{\}$;
$H \leftarrow G$;
$\vec{J} \leftarrow (\underbrace{0, 0, \cdots, 0}_{n \text{ times}})$;
$J[s] \leftarrow -1$;
$J[t] \leftarrow 1$;
$F^{s,t}_{\mathsf{dipole}} \leftarrow$ flows in H due to power injections $\vec{J}$;
for $e \in E$ **do**
  if $F^{s,t}_{\textit{dipole}}(e)F(e) > 0$ **then**
    $E_{\mathsf{be}} \leftarrow E_{\mathsf{be}} \cup \{e\}$;
  else
    $E_{\mathsf{nbe}} \leftarrow E_{\mathsf{nbe}} \cup \{e\}$;
  end
end

The topological predictor

This above algorithm results in a fast numerical way to check for Braessian edges. Now we will expand on this, and construct a *topological* understanding of which edges are Braessian. Note that the problem has now been reduced to understanding this: If a dipole current source is placed across the maxflow edge, what is the direction of flow across an arbitrary edge? If we can answer this, the job is done: The edges where this direction is the same as the original flow, are Braessian.

However this question is hard to answer. There is a purely topological criterion [4] to determine the direction of flows across edge e due to a dipole current source across edge f, but to actually *use* this criterion one needs to iterate over *all simple paths* connecting the endpoints of the edge f that also contain the edge e. This is numerically very expensive, and also does not lead to an intuitive topological understanding of whether an edge is Braessian.

However, by sacrificing perfect accuracy in favour of simplicity, we can design an approximate topological predictor for Braessian edges inspired by the exact topological criterion [4]:

1. Let the maximum flow be across edge (u, v), directed from u to v. Remove the edge (u, v). If (u, v) is a bridge, then there is no Braess paradox in this network.

2. Our aim is to predict if another edge (s, t) is Braessian. The flow there is from s to t.

3. Remove the edge (s, t). If it is a bridge, it is not Braessian.

4. Look at the shortest simple path $[u, \cdots, s, t, \cdots, v]$, i.e. the shortest simple path from u to v that contains the edge (s, t) and touches s before t. Let its length be l^+.

5. Look at the shortest simple path $[u, \cdots, t, s \cdots, v]$. Let its length be l^-.

6. If $l^- < l^+$, predict that (s, t) is Braessian. If $l^- > l^+$, predict that (s, t) is not Braessian. If they are equal, then this predictor does not work (*Alignment undefined* in Figure 3 of the main text).

As before, we refer to [3] for details. We see in Figure 3 of the main text, that the predictor performs quite well.

A note on bridges

If an edge is a *bridge*, i.e. removing it leads to the graph (originally connected) becoming disconnected, then upgrading it leaves the flows across all the edges unchanged. This happens because they are not part of any cycle

and therefore do not support any rerouting flow. Since they are therefore not interesting for the present article, such edges have been intentionally excluded from all the analyses. While generating random network realizations, we have also excluded the ones where the maximum flow is across a bridge.

Supplementary Note 4

Six-node-system displaying Braess' Paradox

The main text discussed the four-node laboratory system, where one line was either modified. Within this Supplementary Information, we have also discussed the case of adding parallel lines (see Supplementary Fig. 8). Complementary to these actions, we could also consider a topological change of the whole network. Specifically, we consider a network of 3 motors and 3 generators, see Supplementary Fig. 13. Line parameters of all lines are, as in the previous experiments, reactance $X = 0.25\Omega$ and resistance $R = 0.18\Omega$. Machines operate at $U \approx 230\,\mathrm{V}$ and $P \approx 1700\,\mathrm{W}$ per phase and $Q \approx 200\,\mathrm{W}$ per phase. These are positive for generators and negative for consumers. These parameters are more symmetric than was achieved in most experiments but give a realistic range for the lab conditions.

We now consider introducing a new line $(2, 5)$, which introduces new cycle flows. These align with the currents on the lines $(3, 4)$ and $(0, 1)$. Using the same load flow calculations as before, the maximum current in the grid is increased by $\Delta I_{\mathrm{relative}} \approx 20\%$ on the two lines $(3, 4)$ and $(0, 1)$, see Table II. This is on the same scale as in the 4 node experiment, where we observed a current increase of up to $\Delta I_{\mathrm{relative}} \approx 25\%$ on one line. This setup shows that a topological alteration to the (test) grid's layout also increases the maximal current in the system, thereby complementing the previous calculations and experiments on upgrading existing lines.

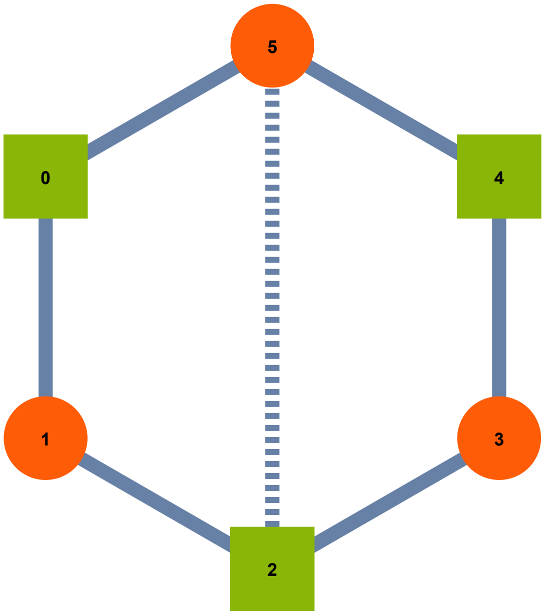

Supplementary Figure 13. Sketch of the 6 node topology investigated: Green squares are generator and red circles consumer nodes. The dashed line is added to the original 6 node network.

Supplementary Table II. Currents before and after the new line $(2, 5)$ is added. We report the absolute value of the current of the original network and the one with the added line. Notably the current on lines $(3, 4)$ and $(0, 1)$ increases by about 20%.

Line	0,1	1,2	2,3	3,4	4,5	5,0	2,5
$I_{\mathrm{Original}}[A]$	3.75	3.70	3.73	3.72	3.71	3.74	-
$I_{\mathrm{Added}}[A]$	4.47	3.10	3.13	4.43	2.95	2.98	1.79
$\Delta I/I_{\mathrm{Original}}$	0.19	-0.16	-0.16	0.19	-0.21	-0.20	-

Supplementary References

[1] Beck, H.-P. & Hesse, R. Virtual synchronous machine. In *Electrical Power Quality and Utilisation, 2007. EPQU 2007. 9th International Conference on*, 1–6 (IEEE, 2007).

[2] Manik, D., Witthaut, D. & Timme, M. Predicting braess' paradox in supply and transport networks. *arXiv preprint arXiv:2205.14685* (2022).

[3] Manik, D. *Dynamics of Complex Flow Networks.* PhD dissertation, Georg-August-Universität Göttingen, freely available at `http://hdl.handle.net/11858/00-1735-0000-002E-E573-8` (2019).

[4] Shapiro, L. W. An electrical lemma. *Mathematics Magazine* **60**, 36–38 (1987).
